# A site-specific risk stratification model for extranodal diffuse large B-cell lymphoma in the oral cavity and maxillofacial region

**DOI:** 10.1007/s00277-026-07029-6

**Published:** 2026-04-29

**Authors:** Yuyang Pang, Yehua Yu, Zhaoli Zhang, Wei Lu, Jun Shi

**Affiliations:** Department of Hematology, Shanghai Ninth People’s Hospital, Shanghai Jiaotong University School of Medicine, Shanghai, China

**Keywords:** Extranodal, Diffuse large B-cell lymphoma, Oral cavity, Maxillofacial region, Prognostic model

## Abstract

**Objective:**

Extranodal diffuse large B-cell lymphoma (DLBCL) of the oral cavity and maxillofacial region (OC-MR) is rare compared to Waldeyer’s ring (WR) DLBCL, characterized by marked aggressiveness and prognostic heterogeneity. This study aims to develop a special risk stratification model for extranodal DLBCL in OC-MR to overcome the limitations of the conventional International Prognostic Index (IPI) to improve outcome prediction in this high-risk population.

**Method:**

We conducted a retrospective analysis of 76 OC-MR DLBCL patients (43 extranodal and 33 WR-DLBCL) diagnosed between January 2015 and March 2022. Survival outcomes were assessed using Kaplan-Meier methodology with log-rank testing. Prognostic factors were identified through Cox regression and LASSO analysis, with model validation via concordance index (C-index).

**Result:**

Patients with extranodal OC-MR DLBCL demonstrated significantly greater multisite involvement (*p* < 0.001) and worse clinical outcomes compared to WR-DLBCL, including shorter median overall survival (17 vs. 26 months, *p* = 0.0329), shorter progression free survival (13 vs. 25 months, *p* = 0.0136) and lower objective response rates (67.4% vs. 84.4%, *p* = 0.003). Hypoalbuminemia (< 37 g/L), elevated C-reactive protein (> 5.83 mg/L), and high Ki67 index (> 80%) predicted poor prognosis. These clinicopathological biomarkers were integrated with IPI to develop the novel exploratory KACIPI model, which demonstrated a potentially improved discriminative ability than IPI (C-index 0.768 vs. 0.680, *p* < 0.001) and effectively stratified extranodal OC-MR DLBCL risk groups (*p* < 0.05).

**Conclusion:**

Extranodal OC-MR DLBCL exhibits distinct clinicopathological features and inferior survival outcomes. The exploratory KACIPI model may provide improved risk prediction for extranodal OC-MR DLBCL by incorporating tumor proliferation, inflammatory markers, nutritional status into conventional IPI score, enabling more accurate identification of high-risk patients.

**Supplementary Information:**

The online version contains supplementary material available at 10.1007/s00277-026-07029-6.

## Introduction

The oral cavity and maxillofacial regions (OC-MR) account for approximately 12% of diffuse large B-cell lymphoma (DLBCL) cases [1], exhibiting remarkable prognostic heterogeneity with 5-year overall survival rates ranging from 45% to 83% [2–4]. According to the Lugano Classification, Waldeyer’s ring (WR) constitutes secondary lymphoid tissue and is thus categorized as a nodal site. WR-DLBCL accounts for the majority (83%) of OC-MR DLBCLs. In contrast, extranodal OC-MR DLBCL which involved in salivary gland, mandible, maxilla, gingiva, oropharyngeal wall and buccal mucosa is relatively rare (17%) [1]. WR-DLBCL typically carries a more favorable prognosis than nodal DLBCL [5–7], whereas extranodal involvement generally portends poorer outcomes [6], with significant variation according to specific anatomical sites [5, 8–12]. Recent data highlight this site-specific variability, showing 5-year survival rates of 63.6% for salivary gland involvement versus 38.2% for oral cavity lesions [10]. This prognostic diversity underscores the need for precise risk stratification in extranodal OC-MR DLBCL.

The International Prognostic Index (IPI), incorporating age, Eastern Cooperative Oncology Group (ECOG) performance status, Ann Arbor stage, lactate dehydrogenase (LDH) levels, and extranodal involvement, remains the cornerstone prognostic tool for DLBCL [13–15]. However, its generalized design fails to account for the biological diversity of DLBCL subtypes, as evidenced by significant outcome variations among patients with identical IPI scores [16]. Particularly in extranodal DLBCL including central nervous system (CNS), gastrointestinal, and testicular presentations, the IPI demonstrates limited prognostic accuracy [17–19]. Similarly, in primary sinonasal DLBCL, prognostic heterogeneity is overwhelmingly driven by molecular subtype (cell-of-origin) rather than the IPI factors [20]. This underscores that for extranodal lymphomas, a refined prognostic model must integrate critical biological determinants like molecular subtype, alongside clinicopathological factors. Current staging systems such as Ann Arbor and Lugano classifications showed limited prognostic utility for extranodal DLBCL affecting specific anatomical sites including intestinal, breast, skin, and leg [16, 21]. The IPI focuses primarily on tumor burden and performance status through its five clinical parameters, yet neglects essential elements such as pathological characteristics, tumor microenvironment features, and particularly host immune and inflammatory responses. Furthermore, emerging evidence suggests that aggressive extranodal DLBCLs at various sites (including sinonasal, skin, breast, and CNS) may share common molecular features (e.g., ABC/MCD subtype with MYD88/CD79B mutations), potentially explaining their distinct dissemination patterns [22]. Consequently, the biological heterogeneity of extranodal OC-MR DLBCL and the recognized limitations of the IPI system necessitate the development of an optimized prognostic model specifically validated for this patient population. Emerging biomarkers including serum inflammatory markers, Ki67 proliferation index, and cell-of-origin classification show prognostic potential in extranodal DLBCL [23–28]. However, their role in extranodal OC-MR DLBCL requires validation.

In this study, the clinical data of extranodal OC-MR DLBCL was retrospectively analyzed and risk factors beyond IPI parameters were then identified by Cox analysis. Furthermore, a novel prognostic risk model was developed to improve the risk stratification of extranodal OC-MR DLBCL.

## Materials and methods

### Patients

We conducted a retrospective study of 76 DLBCL patients of OC-MR treated at our institution between January 2015 and March 2022. Diagnosis was confirmed according to the 2016 WHO classification of hematopoietic and lymphoid tumors. Eligible patients met the following criteria: (1) Histologically confirmed DLBCL. (2) Availability of complete baseline clinical data. (3) Sufficient follow-up for endpoint assessment: Patients were required to be followed until death, disease progression/relapse, or for a minimum of 12 months post-diagnosis if they were still alive and progression-free at the data cutoff. (4) The primary symptom appeared in OC-MR and patients received standard treatment, including immunochemotherapy, local radiation, targeted therapies, and autologous hematopoietic stem cell transplantation. The cohort comprised 43 extranodal cases (salivary glands, gingiva, hard palate, mandible/maxilla) and 33 WR cases (tonsils, oropharynx, nasopharynx, tongue base and soft palate). Primary maxillary lymphomas were defined as those presenting with initial symptoms and bulk of disease in maxillary structures, without evidence of primary sinonasal origin. Cases with clear sinonasal primary with secondary maxillary extension were excluded based on radiographic and clinical presentation review. This retrospective study was approved by the Institutional Review Board of Shanghai Ninth People’s Hospital, Shanghai Jiao Tong University School of Medicine (Approval No. SH9H-2023-T193-1) with waiver of informed consent, in accordance with the Declaration of Helsinki. 

### Treatment and response assessment

All patients received R‑CHOP (rituximab, cyclophosphamide, doxorubicin, vincristine, and prednisone) based immunochemotherapy, with subgroups receiving additional immunomodulators/BTK inhibitors, radiotherapy, or autologous hematopoietic stem cell transplantation. Treatment response was evaluated according to the 2014 Lugano classification criteria (NCCN guidelines), categorizing outcomes as complete response (CR; resolution of all detectable disease), partial response (PR; ≥ 50% reduction in measurable lesions), stable disease (SD; failure to meet CR/PR criteria without progression), or progressive disease (PD; ≥ 50% increase in lesions or new involvement), with objective response rate (ORR) defined as the proportion of patients achieving CR and PR.

### Study endpoints

The primary endpoint was overall survival (OS), defined as the time from diagnosis to death from any cause. The secondary endpoints were progression-free survival (PFS), defined as the time from diagnosis to the first occurrence of disease progression, relapse, or death from any cause and ORR.

### Determination of biomarker cut-off values

Optimal prognostic cut-offs for the continuous variables were determined using time-dependent receiver operating characteristic (t-ROC) curve analysis with a 36-month horizon. This method appropriately accounts for censored observations and aligns with the validation framework applied later in the manuscript. The cut-off value that maximized Youden’s index (sensitivity + specificity – 1) was selected for each biomarker.

### Construction and confirmation of the forecast model

The prognostic relevance of the candidate prognostic factors was evaluated using univariate Cox regression for OS. The least absolute shrinkage and selection operator (LASSO) Cox regression analysis was employed to analyze the prognosis-related factors. Subsequently, the variability in overall persistence across these two classes was examined using Kaplan-Meier curves as well as a log-rank test. Ultimately, the time-dependent ROC curve examination was employed to determine the specificity and sensitivity of the risk signature utilizing the “survival ROC” R package. The predictive accuracy and discriminability of the novel risk score were validated through the area under the curve (AUC), concordance index (C-index) and calibration plots.

### Statistical analysis

The analysis was performed on a complete dataset. All variables used for model development and validation (including diagnostic pathology, IPI factors, and laboratory biomarkers) had no missing values, as verified by a pre-analysis audit of the database. Statistical analysis was performed using SPSS Statistics version 28 (IBM Corp., Armonk, NY, USA), R software version 4.2.1 (R Foundation for Statistical Computing, Vienna, Austria), and GraphPad Prism 8 (GraphPad Software, San Diego, CA, USA). Categorical variables were analyzed using χ² tests or Fisher’s exact tests, as appropriate. Continuous variables were presented as mean ± standard deviation. OS was estimated using the Kaplan-Meier method, with between-group comparisons performed using log-rank (Mantel-Cox) tests. The proportional hazards assumption for the Cox regression models was verified graphically using log-minus-log (LML) survival plots. A two-sided *p*-value < 0.05 was considered statistically significant.

## Results

### Clinical characteristics of extranodal OC-MR DLBCL patients

The extranodal OC-MR DLBCL cohort (*n* = 43) demonstrated a median age of 63 years (range: 28–79; *p* = 0.264 for comparison with WR group) with male predominance (79.1%; *p* = 0.075). Clinically, 51.2% presented with localized disease (Ann Arbor stage I/II; *p* = 0.412), while IPI stratification revealed 62.8% (28/43) in low/low-intermediate risk categories (*p* = 0.904). Most patients (86%, 37/43) had preserved performance status (ECOG 0; *p* = 0.148). The extranodal group was predominantly composed of the germinal center B-cell (GCB) subtype (55.8%; *p* = 0.217 vs. WR group), with the non-GCB subtype accounting for the remainder (44.2%). Comparative analysis showed significantly greater frequency of multisite extranodal involvement (≥ 2 extranodal sites) in extranodal versus WR-DLBCL (*p* < 0.001). However, no other baseline characteristics, including treatment regimens (Supplement Table 1. *p* > 0.05), indicated statistically significant differences between groups (Table 1).

**Table 1:** Baseline characteristics

Characteristics	WR-DLBCL (*n* = 33)	Extranodal OC-MR DLBCL (*n* = 43)	*P* value
Gender, n (%)			0.075
Male	19 (57.5)	34 (79.1)	
Female	14 (42.5)	9 (22.9)	
Age (years), n (%)			0.264
≤ 60	9 (27.3)	17 (20.1)	
> 60	24 (72.7)	26 (79.9)	
Ann Arbor stage, n (%)			0.412
I/II	20 (60.6)	22 (51.2)	
III/IV	13 (39.4)	21 (48.8)	
Extranodal involvement, n (%)			< 0.001
< 2	26 (78.8)	17 (39.5)	
≥ 2	7 (21.2)	26 (60.5)	
Serum LDH level			0.484
< ULN	22(66.7)	25(58.1)	
≥ ULN	11(33.3)	18(41.9)	
ECOG-PS, n (%)			0.148
< 2	24 (72.7)	37 (86.0)	
≥ 2	9 (27.3)	6 (14.0)	
IPI, n (%)			0.904
Low risk (0–1)	14 (42.4)	16 (37.2)	
Low-intermediate risk (2)	9 (27.3)	11 (25.6)	
High-intermediate risk (3)	4 (12.1)	6 (14.0)	
High risk(4–5)	6 (18.2)	10 (23.2)	
Han’s classification, n (%)			0.217
GCB	10 (30.3)	19 (44.2)	
non-GCB	23 (69.7)	24 (55.8)	

*Abbreviations* *OC-MR* oral cavity-maxillofacial region, *DLBCL* Diffuse large B-cell lymphoma, *WR* Wechsler's ring, *NWR* non-Wechsler's ring, *ECOG* Eastern Cooperative Oncology Group, PS performance status, *IPI* International Prognostic Index, *LDH* lactate dehydrogenase, *ULN* upper limit of normal

NWR-DLBCL denotes OC-MR-localized extranodal disease distinct from Waldeyer's ring involvement

### The survival outcomes of extranodal OC-MR DLBCL patients were poor

**Fig. 1:**
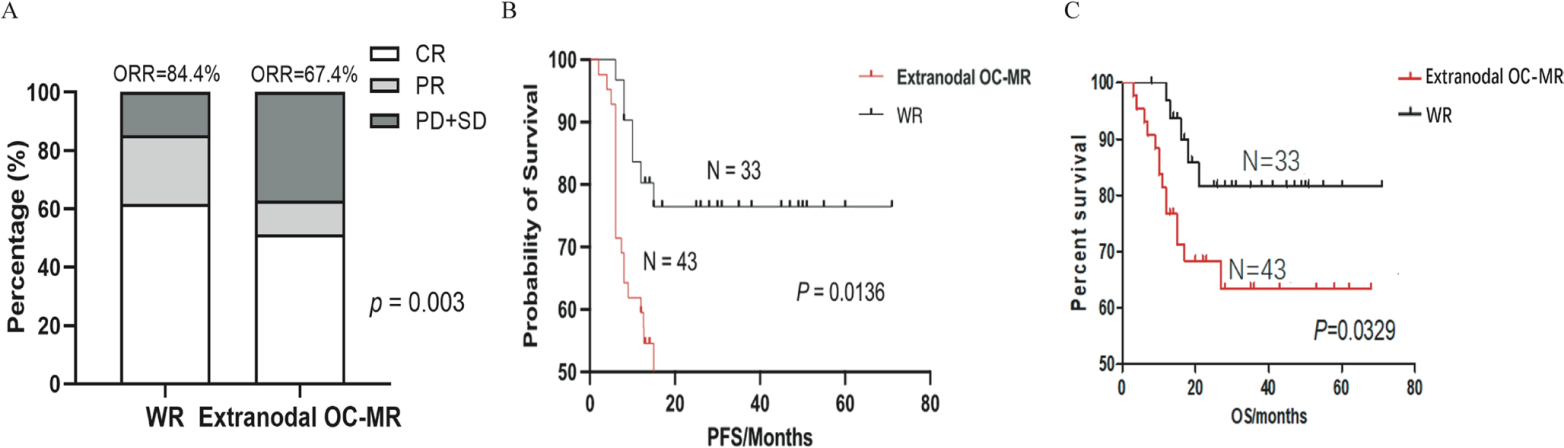
Comparative treatment response and survival outcomes between extranodal OC-MR DLBCL and WR-DLBCL cohorts. (**A**) ORR analysis showing significantly lower CR/PR rates in extranodal OC-MR DLBCL (67.4%) compared to WR-DLBCL (84.4%; *p* = 0.003). (**B**) PFS was significantly shorter in extranodal cases (*p* = 0.0136). (**C**) OS analysis revealing significantly inferior outcomes in extranodal OC-MR DLBCL (*n* = 43, 14 deaths) versus WR-DLBCL (*n* = 33, 5 deaths; *p* = 0.0329). ORR, objective response rate; OS, overall survival; PFS, progression-free survival; PR, partial response; PD, stable disease; SD, progressive disease

Building on these clinical characteristics, we observed significantly poorer outcomes in extranodal OC-MR DLBCL. The ORR was significantly lower in extranodal OC-MR DLBCL patients compared to WR-DLBCL patients (62.7% vs. 85.3%, *p* = 0.003, Fig. 1A). After a median follow-up of 26 months (range: 3–68 months), PFS was significantly shorter in extranodal cases (median PFS: 13 vs. 25 months, *p* = 0.0136, Fig. 1B), consistent with the inferior OS (median OS: 17 vs. 26 months, *p* = 0.0329, Fig. 1C) observed in these patients. These findings collectively indicated that the reduced survival in extranodal OC-MR DLBCL primarily results from intrinsic treatment refractoriness rather than differential relapse patterns, underscoring the need for biomarker discovery to guide therapeutic strategies in this high-risk population.

### Evaluation of the IPI for risk stratification in extranodal OC-MR DLBCL patients

**Fig. 2:**
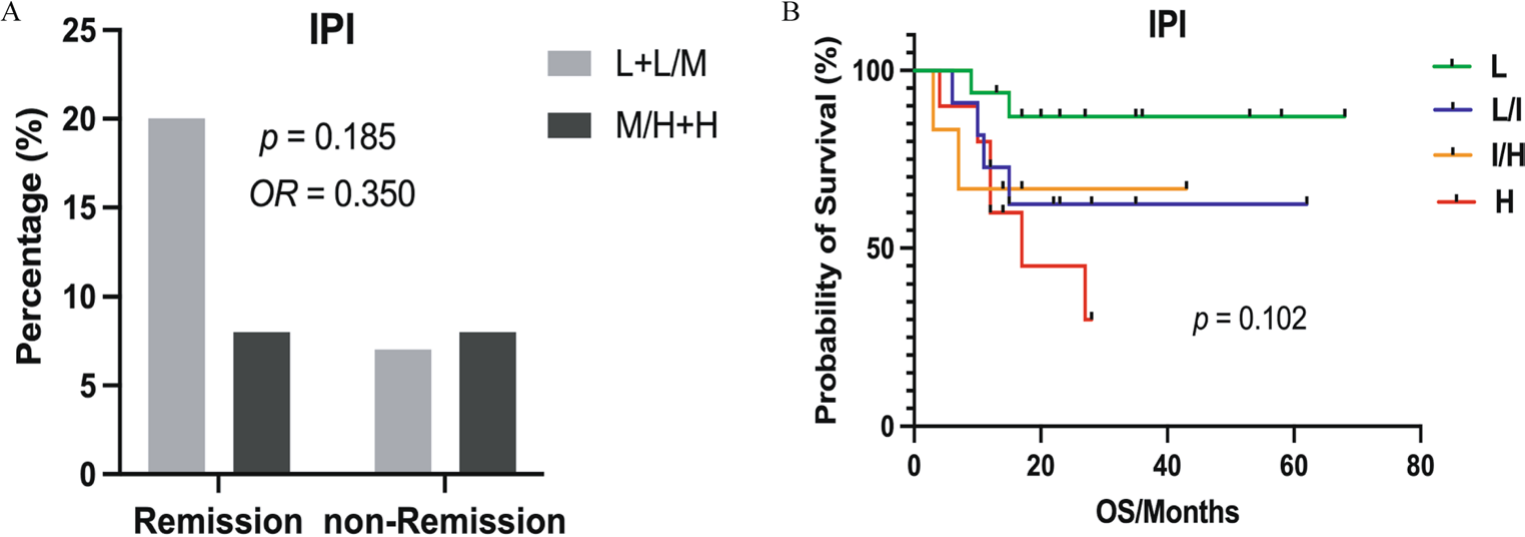
Treatment response and survival outcomes in extranodal OC-MR DLBCL patients stratified by IPI. (**A**) ORR analysis across IPI risk groups. No statistically significant difference was observed (*p* = 0.185). (**B**) Kaplan-Meier survival curves comparing OS between patients achieving remission versus non-remission status (*p* = 0.102). The x-axis represents follow-up time in months, and the y-axis shows probability of survival (%). L, low risk; L/M, low-intermediate risk; M/H, intermediate/high risk; H, high risk; OR, odds ratio

Considering the widespread application of the IPI scoring system in DLBCL, it was evaluated in extranodal OC-MR DLBCL patients. Comparative analysis revealed significant differences in OS and ORR between extranodal and WR-DLBCL groups despite comparable IPI distributions. In the extranodal cohort, the IPI showed an association with OS (*p* = 0.011, Fig. 2A). However, it showed limited ability to separate patients into the four distinct risk categories (*p* = 0.102, Fig. 2B). These findings raise the possibility that conventional IPI scoring may have limitations in extranodal OC-MR DLBCL and suggest that other prognostic factors deserve further investigation in this patient population.

### Spectrum and frequency of involved anatomical sites in extranodal OC-MR DLBCL

To further explore prognostic determinants, we analyzed the anatomical spectrum of tumor involvement in extranodal OC-MR DLBCL cohort, the most involved anatomical sites included the maxillary and mandibular regions (48.8%), salivary glands (parotid, submandibular, and sublingual glands; 18.6%), gingiva (18.6%), palatal region (14.0%), and other sites (tongue, masticatory, and facial muscles; 7.0%). Among the 43 extranodal OC-MR DLBCL cases, 26 involved two or more extranodal sites. The involvement patterns comprised multifocal OC-MR disease (*n* = 7), extension to adjacent head and neck sites (*n* = 7), and distant dissemination (*n* = 12). Of the patients with distant dissemination, the most frequently involved sites were abdominal organs (14.49%), followed by bone (13.04%), bone marrow (11.59%), reproductive organs (7.25%), heart/lung (5.80%), skin (5.80%), muscle (2.90%), and the central nervous system (2.90%). Despite a higher frequency of multisite involvement (≥ 2 sites) in extranodal OC-MR DLBCL, this characteristic was not significantly associated with OS (*p* = 0.145).

### Two clinical biomarkers were identified as prognostic factors for extranodal OC-MR DLBCL patients

Clinical biomarkers were given more attention to analyze their effect on the prognosis of DLBCL, they were also investigated in extranodal OC-MR DLBCL patients in this study. Through comprehensive evaluation of clinical prognostic markers, we identified three significant biomarkers in extranodal OC-MR DLBCL. It should be noted that the optimal cutoff values were derived from ROC curve analysis using OS status as the classification variable, and these cutoffs may show instability particularly in limited sample sizes. First, ROC curve analysis determined optimal cut-off values: albumin (ALB) < 37 g/L (AUC = 0.724, *p* = 0.018), β2-microglobulin (B2MG) > 2.18 mg/L (AUC = 0.694, *p* = 0.038), and C-reactive protein (CRP) > 5.83 mg/L (AUC = 0.742, *p* = 0.009) (Supplementary Fig. 1A-C). Then, univariate Cox analysis identified ALB < 37 g/L, B2MG > 2.18 mg/L, and CRP > 5.83 mg/L as significant predictors of both inferior OS (all *p* < 0.05) and reduced ORR (Table 2). Additionally, B2MG > 2.18 mg/L and CRP > 5.83 mg/L were also significantly associated with inferior PFS (both *p* < 0.05). A trend toward worse PFS was also observed for low albumin levels (< 37 g/L), though this association did not reach formal statistical significance (*p* = 0.055). B2MG was excluded from the final model due to its imprecise effect estimate (wide confidence interval) and to mitigate overfitting risk given the limited event count (*n* = 14). We prioritized more robust and clinically practical biomarkers, establishing albumin and CRP as clinically relevant for risk stratification in extranodal OC-MR DLBCL.

**Table 2:** Univariate analysis of clinical and pathological prognostic factors in extranodal OC-MR DLBCL

Variables	ORR			PFS			OS		
	*P*	OR	95%CI	*P*	HR	95%CI	*P*	HR	95%CI
IPI	0.188	2.714	0.727–8.924	0.003	1.761	1.216–2.549	0.021	1.672	1.087–2.192
ALB (g/L) < 37 v ≥ 37	0.015	0.146	0.041–0.676	0.055	2.317	0.984–5.456	0.010	4.047	1.394–11.752
β2-MG (mg/L) > 2.18 v ≤ 2.18	0.006	0.071	0.006–0.558	0.008	5.307	1.553–18.131	0.041	8.356	1.091–64.015
CRP (mg/L) > 5.83 v ≤ 5.83	0.019	0.205	0.047–0.717	0.006	3.331	1.403–7.907	0.017	3.806	1.271–11.388
Ki67 > 80% v ≤ 80%	0.019	0.137	0.034–0.599	0.014	3.043	1.249–7.412	0.003	5.05	1.143–11.743
Han’s classification non-GCB v GCB	0.755	0.769	0.236–2.652	0.416	1.435	0.601–3.428	0.878	1.086	0.376–3.138
DEL v non-DEL	0.215	0.330	0.075–1.438	0.057	2.517	0.972–6.513	0.181	2.210	0.692–7.060
CD5 + v CD5-	0.419	0.458	0.118 to 1.844	0.301	1.702	0.622–4.656	0.180	2.242	0.689–7.290

*Abbreviations*: *ALB* Albumin, *β2-MG* β2-microglobulin, *CRP* C-reactive protein, *DEL* double-expressor lymphoma, *non-GCB* non-Germinal Center B-cell like, *GCB* Germinal Center B-cell-like

### High Ki67 index was poor for the prognosis of extranodal OC-MR DLBCL patients

Since the prognosis of DLBCL was influenced by cell origin and molecular expression in immunohistochemical, pathological characteristics were further explored in extranodal OC-MR DLBCL patients. Pathologically, we evaluated several immunohistochemical markers (Ki67, Han’s classification, double-expressor lymphoma phenotype, and CD5 expression) through univariate Cox regression analysis. ROC curve analysis established Ki67 > 80% as a significant prognostic cut-off value (AUC 0.736, *p* = 0.013) (Supplementary Fig. 1D). Elevated Ki67 (> 80%) was associated with inferior OS (*p* = 0.003) and PFS (*p* = 0.014), as well as a reduced ORR (*p* = 0.019). The double-expressor lymphoma (DEL) phenotype demonstrated a trend toward worse PFS (*p* = 0.057). No significant associations were observed with OS (*p* = 0.181) or ORR (*p* = 0.215) (Table 2). Notably, we identified a significant positive correlation between Ki67 > 80% and DEL status (Odds Ratio = 9.429; *p* = 0.038), which may reflect an underlying biological interaction. In contrast, neither Han’s classification (GCB vs. non-GCB) nor CD5 expression showed significant prognostic value for any clinical endpoints (all *p* > 0.05, Table 2). These findings suggest the distinct prognostic roles of proliferative activity (Ki67) and DEL phenotype in extranodal OC-MR DLBCL, while observing limited utility of conventional pathological markers in this anatomical subset.

### The exploratory KACIPI model for prognostic stratification in extranodal OC-MR DLBCL patients.

**Fig. 3:**
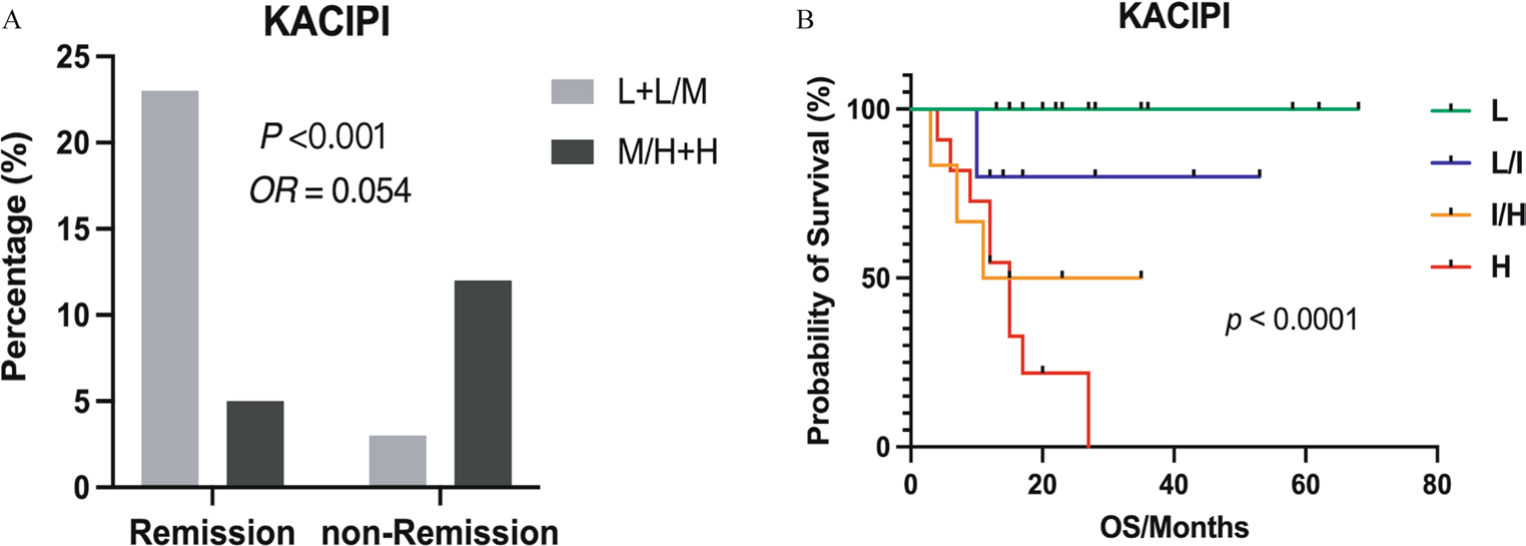
Treatment response and survival outcomes in extranodal OC-MR DLBCL patients stratified by KACIPI score. (**A**) The KACIPI score showed a strong negative association with ORR (OR = 0.054, *p* < 0.001). (**B**) Kaplan-Meier analysis revealed significant stratification of OS across KACIPI risk categories (*p* < 0.001). KACIPI, Ki67-Albumin-CRP-Enhanced International Prognostic Index

Within the extranodal OC-MR DLBCL cohort, the individual variables Ki67 (≥ 80%), hypoalbuminemia (< 37 g/L), and elevated CRP (> 5.83 mg/L) each demonstrated strong and consistent associations with inferior OS (all *p* < 0.01). Prior to model construction, we validated the proportional hazards assumption for all candidate variables. As illustrated in Supplementary Fig. 2, the LML survival plots for Ki67, albumin, CRP and IPI, revealed approximately parallel curves across strata, confirming the appropriateness of the Cox regression framework. Building on these validated predictors, we developed the exploratory KACIPI prognostic model by incorporating Ki67 > 80%, albumin < 37 g/L, and CRP > 5.83 mg/L into the conventional IPI framework. Using multivariate Cox regression coefficients, the KACIPI risk score was calculated as: 0.139×IPI + 1.196×Ki67 (if > 80%) + 0.898×ALB (if < 37 g/L) + 0.378×CRP (if > 5.83 mg/L), where each variable was coded as present (1) or absent (0). Patients were stratified into four distinct prognostic groups by quartile: low-risk (0-0.272), low-intermediate-risk (0.273–0.817), intermediate-high-risk (0.818–1.558), and high-risk (> 1.558). In an exploratory analysis confined to the extranodal OC-MR DLBCL cohort, the KACIPI model showed improved predictive accuracy for ORR relative to the conventional IPI (OR = 0.054, *p* < 0.001, Fig. 3A). For the primary endpoint of OS, the model stratified patients into four risk categories with distinct outcomes (*p* < 0.001, Fig. 3B). These biomarkers maintained independent prognostic value beyond conventional IPI parameters (LDH, disease stage, or ≥ 2 extranodal sites; all *p* > 0.05), while demonstrating specificity for extranodal OC-MR DLBCL through their lack of prognostic significance in WR-DLBCL cohort (Supplementary Table 2). This refined risk stratification highlights the clinical utility of incorporating tumor proliferative activity (Ki67), nutritional status (albumin), and systemic inflammation (CRP) into prognostic models for extranodal OC-MR DLBCL.

### Development and validation of the KACIPI prognostic model via LASSO regression

**Fig. 4:**
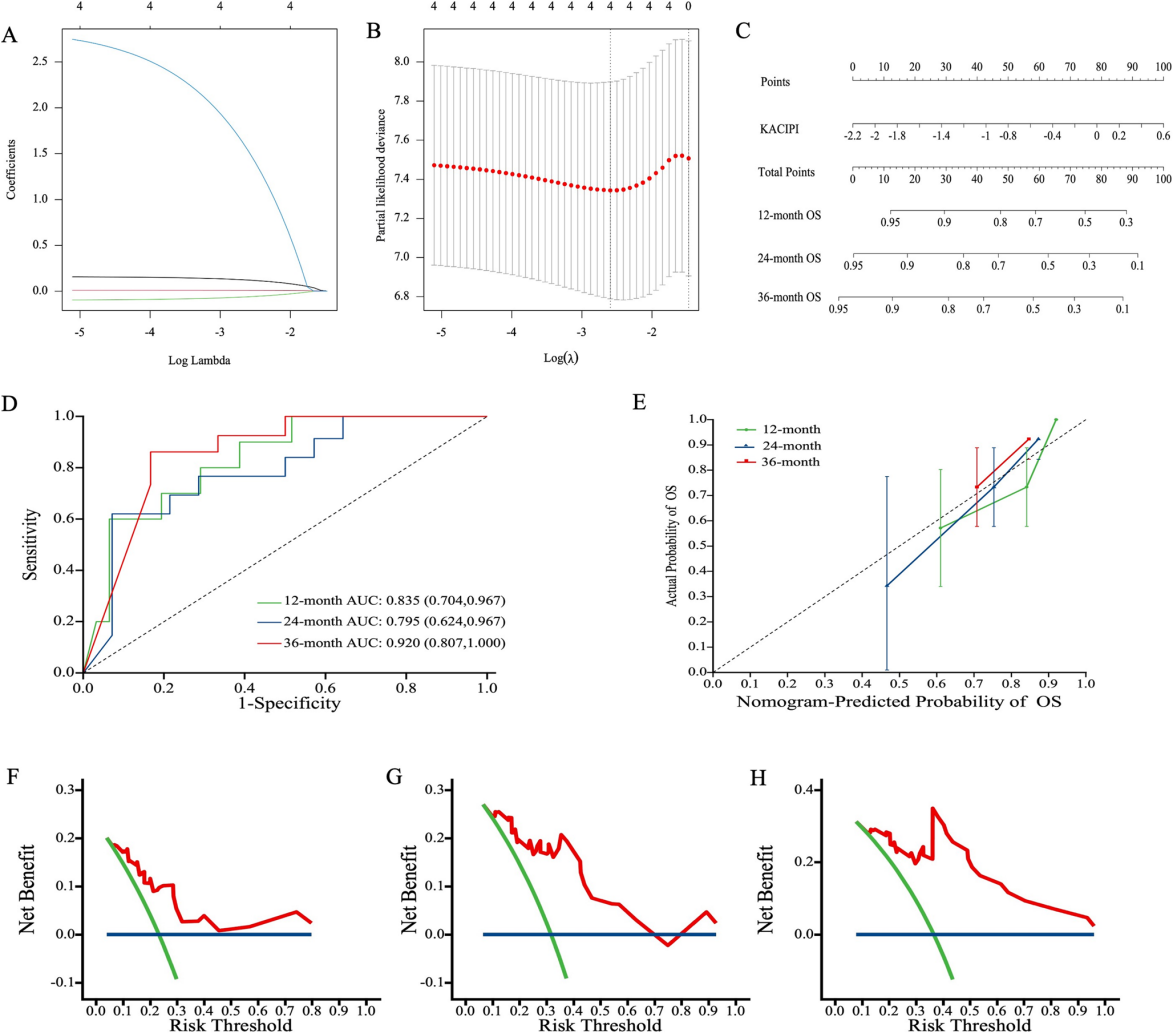
Development and validation of the KACIPI prognostic model for extranodal OC-MR DLBCL patients using LASSO regression analysis. (**A**) LASSO regression coefficients for the four selected variables, determined through 10-fold cross-validation. (**B**) Partial likelihood deviance curve derived from the LASSO model, with optimal lambda (λ) values identified via 10-fold cross-validation. (**C**) Clinical nomogram predicting 36-month OS probability based on the KACIPI score. LASSO, least absolute shrinkage, and selection operator. (**D**) Time-dependent ROC analysis showing predictive accuracy (tdAUROC) over follow-up. (**E**) Calibration plots comparing predicted vs. observed OS. Visual assessment indicates reasonable calibration across the range of predicted risk. (**F**) Decision curve analysis for 1-year OS. (**G**) Decision curve analysis for 2-year OS. (**H**) Decision curve analysis for 3-year OS

To objectively validate the KACIPI model, we performed LASSO regression incorporating Ki67, ALB, CRP, and IPI score, yielding the final model: 0.120×IPI + 0.009×CRP − 0.061×ALB + 1.523×Ki67 (Fig. 4A-B). A nomogram was constructed based on the risk scores and total points to predict individualized prognosis in extranodal OC-MR DLBCL patients, providing a user-friendly tool for clinical application (Fig. 4C). Time-dependent ROC analysis confirmed sustained predictive accuracy of the risk score for OS across follow-up periods with AUC values of 0.835 (95% CI, 0.704–0.967) at 1 year, 0.795 (95% CI, 0.624–0.967) at 2 years, and 0.920 (95% CI, 0.807-1.000) at 3 years (Figs. 4D). The model demonstrated superior discriminative ability (C-index 0.768, 95% CI, 0.670–0.866) versus IPI (0.680, 95% CI, 0.564–0.796). The calibration curve, constructed using bootstrap resampling validation, showed reasonable visual agreement between predicted and observed survival probabilities (Fig. 4E). The decision curve analysis demonstrated a positive net benefit for predicting 1-, 2-, and 3-year overall survival (OS) across clinically relevant threshold probabilities, indicating the model’s potential utility in clinical decision-making (Figs. 4F-H). These validation results confirmed the KACIPI model as a clinically useful prognostic system that demonstrates improved performance over the conventional IPI for risk stratification in extranodal OC-MR DLBCL.

## Discussion

Our study establishes extranodal OC-MR DLBCL as a distinct aggressive subtype with poorer OS and ORR than WR DLBCL, independent of treatment regimens. This phenotype highlights the need for specialized prognostic tools. We identified three OC-MR-specific risk factors: Ki67 > 80%, ALB < 37 g/L, and CRP > 5.83 mg/L, reflecting tumor proliferation, nutrition, and inflammation. The exploratory KACIPI model incorporating these readily available markers with IPI showed superior prognostic performance, effectively stratifying patients into four distinct risk groups. These suggest that the KACIPI may serve as a clinically useful prognostic tool for extranodal OC-MR DLBCL.

The IPI demonstrated limited prognostic utility in extranodal OC-MR DLBCL, failing to effectively stratify risk in our cohort, consistent with observations in other extranodal sites including CNS, gastrointestinal and testicular DLBCL [17–19]. This limitation stems from the IPI’s failure to account for distinctive extranodal disease biology, particularly microenvironmental influences and host inflammatory responses.

Our study has identified several novel prognostic factors that redefine our understanding of extranodal OC-MR DLBCL. Clinically, we confirmed that hypoalbuminemia (< 37 g/L) serves as a composite marker of nutritional status and systemic inflammation, with its adverse prognostic impact validated in primary central nervous system lymphoma (PCNSL) and gastric DLBCL [29, 30]. Elevated CRP (> 5.83 mg/L) robustly predicts disease progression, mirroring its established role as a negative prognostic indicator in PCNSL [29] and other DLBCL variants [31, 32]. These biomarkers collectively capture critical aspects of tumor biology and host response that are particularly relevant in extranodal OC-MR DLBCL.

Pathologically, our study establishes a Ki67 cutoff of > 80% for extranodal OC-MR DLBCL, higher than the standard 70% threshold for nodal DLBCL [3, 5], yet consistent with known prognostic patterns [33]. This elevated threshold suggests extranodal OC-MR DLBCL exhibits particularly aggressive proliferation characteristics. The strong association between Ki67 > 80% and DEL status aligns with previous reports of DEL cases showing higher proliferation indices [34]. This relationship may reflect combined effects of MYC-driven cell cycle activation and BCL2-mediated survival advantages. The link between DEL/high-Ki67 and reduced T-cell infiltration [35] further suggests important tumor microenvironment interactions. In our cohort, DEL was associated with worse PFS. It is important to note that DEL cases are predominantly of the non-GCB (particularly MCD) subtype; therefore, the observed correlation between DEL and worse PFS may, in part, reflect the underlying aggressive cell-of-origin biology rather than an independent effect of the double-expressor phenotype itself. While some studies report oral DLBCL frequently shows non-GCB subtype with elevated Ki67 [36, 37] and CD5 + cases often demonstrate aggressive features [25, 38], our findings differ. In our cohort of extranodal OC-MR DLBCL, the prognostic associations for Han’s classification and CD5 expression did not reach statistical significance, and no link with high Ki67 was found. This observation, however, does not preclude the potential importance of molecular subtypes (e.g., MCD) that are not captured by the Hans algorithm. In sinonasal DLBCL, molecular subtype was the primary determinant of outcome, with the GCB subtype showing minimal mortality [20]. The distinct recurrence patterns observed in some patients may be linked to the ABC/MCD subtype, which is shared among aggressive extranodal DLBCLs across various anatomical sites [20, 22]. Our clinical model does not account for molecular heterogeneity. Future models must integrate molecular subtyping to enable precise risk stratification and guide targeted therapies.

Notably, these biomarkers maintained independent prognostic value beyond conventional parameters including LDH levels and Ann Arbor stage, while demonstrating specificity for extranodal OC-MR DLBCL through their lack of prognostic significance in WR-DLBCL. The clinical relevance of these markers is well-supported by existing literature, with studies showing that CRP or Ki67 incorporation enhances predictive accuracy in nodal DLBCL [39–41], albumin-containing models improve prognostic stratification across both nodal [42–44] and extranodal DLBCL variants including PCNSL and gastrointestinal (GI)-DLBCL [17, 45], and integration of these markers with IPI significantly strengthens risk prediction capabilities [17, 46].

Our findings carry significant clinical implications that warrant attention. First and foremost, extranodal OC-MR DLBCL should be formally recognized as a distinct clinical entity requiring specialized risk assessment protocols. The newly developed KACIPI model provides clinicians with a practical and validated tool that holds promise for accurate risk stratification and clinical decision-making. Future research efforts should prioritize three critical areas including prospective multicenter validation of the KACIPI model in larger patient cohorts, in-depth investigation of the biological mechanisms driving treatment resistance in extranodal OC-MR DLBCL, and the development of tailored treatment strategies specifically designed for high-risk patients identified through KACIPI scoring.

Our study has several important limitations. First, the sample size of the extranodal cohort is modest, which constrains the robustness of any multivariable prognostic model and increases the risk of overfitting. Therefore, the KACIPI score should be viewed as exploratory and hypothesis-generating rather than a validated clinical tool. Its primary value lies in highlighting the combined prognostic role of tumor proliferation (Ki67), nutritional status (albumin), and systemic inflammation (CRP). Second, due to the limited number of patients within each specific anatomical subsite (e.g., salivary gland, gingiva, palate), a statistically meaningful analysis of prognosis by exact location could not be performed. Third, our model used the Hans immunohistochemical classification and did not include genetic subtyping, which carries distinct prognostic information not captured by Han’s. Future studies should integrate molecular profiling for more precise stratification. Fourth, the calibration of the prognostic model was evaluated visually. Future validation in larger cohorts should incorporate quantitative metrics such as the calibration slope, intercept, and Brier score to provide a more rigorous assessment of model fit. Future collaborative, multicenter studies will be required to investigate potential outcome differences based on precise anatomical origin and molecular subtype within the OC-MR region.

In summary, our results revealed that the prognosis of extranodal OC-MR DLBCL patients is worse than that of WR-DLBCL. An exploratory prognostic model combined IPI with Ki67 index, CRP and ALB maybe more suitable for the prognosis stratification of extranodal OC-MR DLBCL than IPI score. Furthermore, the KACIPI score, which integrates routine clinical data, serves as a hypothesis-generating tool. In the context of contemporary immunochemotherapy, it may help to stratify patients according to progression risk, potentially guiding future research into risk-adapted therapeutic approaches.

## Supplementary Material

Supplementary Material 1. ROC curve and cut-off values of prognostic indicators in extranodal OC-MR DLBCL patients. (**A**) ROC curve and cut-off value of ALB. (**B**) ROC curve and cut-off value of β2-MG. (**C**) ROC curve and cut-off value of CRP. (**D**) ROC curve and cut-off value of Ki67.

Supplementary Material 2.

Supplementary Material 3.

Supplementary Material 4. LML plots for the variables in the KAC-IPI model. The approximate parallelism of the curves indicates that the proportional hazards assumption was met for (**A**) IPI, (**B**) Ki67, (**C**) CRP, and (**D**) Albumin.

## Data Availability

All data examined during this study can be obtained from public databases.
