# Supplementary figures and images for: A site-specific risk stratification model for extranodal diffuse large B-cell lymphoma in the oral cavity and maxillofacial region
Source: Ann Hematol. 2026 Apr 29;105(5):272. doi: 10.1007/s00277-026-07029-6 (PMC13128735; doi:10.1007/s00277-026-07029-6)

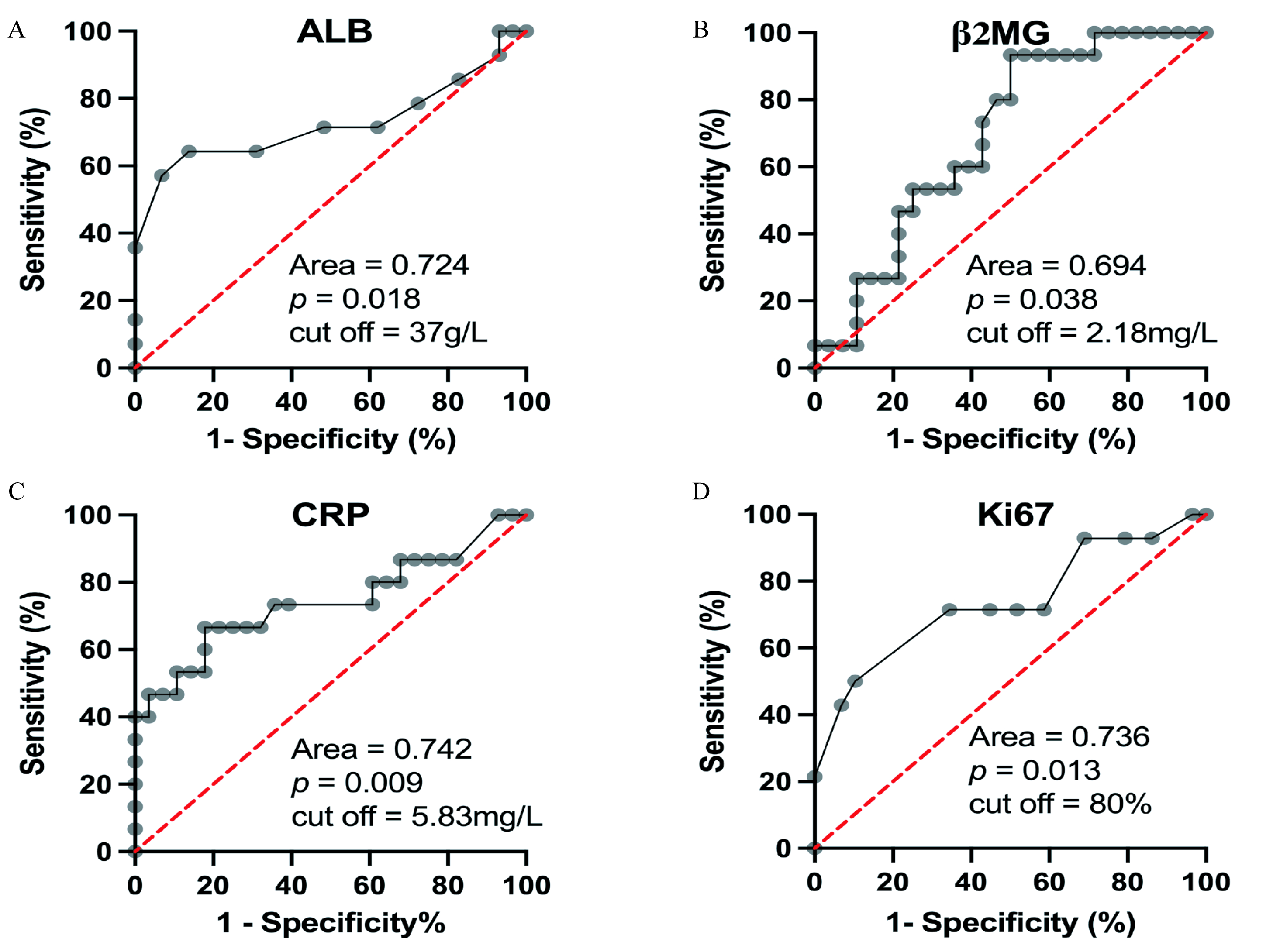

Supplement: Supplementary file 1 — Supplementary Material 1. ROC curve and cut-off values of prognostic indicators in extranodal OC-MR DLBCL patients. (A) ROC curve and cut-off value of ALB. (B) ROC curve and cut-off value of β2-MG. (C) ROC curve and cut-off value of CRP. (D) ROC curve and cut-off value of Ki67. [file 277_2026_7029_MOESM1_ESM.tif]

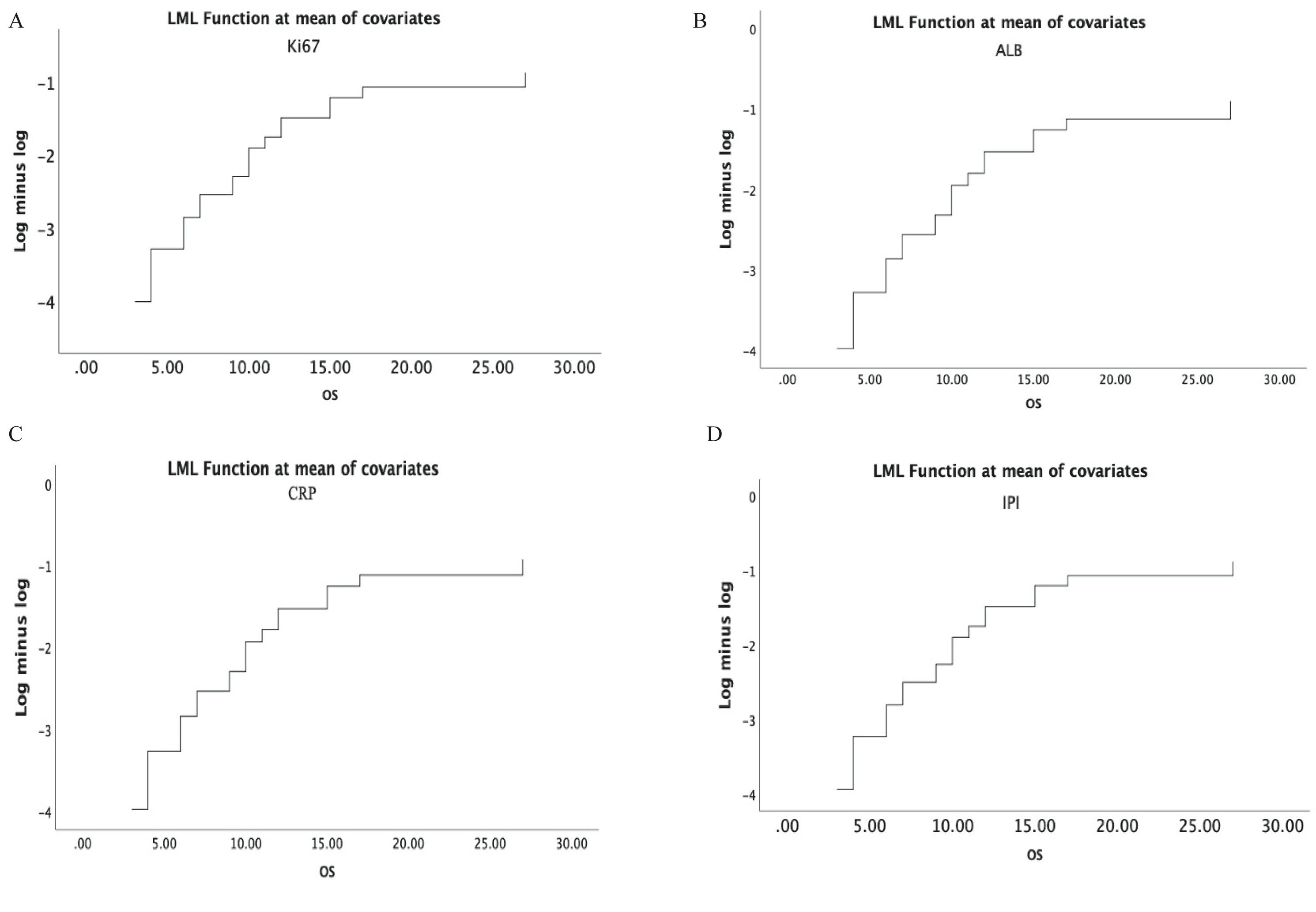

Supplement: Supplementary file 4 — Supplementary Material 4. LML plots for the variables in the KAC-IPI model. The approximate parallelism of the curves indicates that the proportional hazards assumption was met for (A) IPI, (B) Ki67, (C) CRP, and (D) Albumin. [file 277_2026_7029_MOESM4_ESM.jpg]
