# Supplementary material for: A site-specific risk stratification model for extranodal diffuse large B-cell lymphoma in the oral cavity and maxillofacial region
Source: Ann Hematol. 2026 Apr 29;105(5):272. doi: 10.1007/s00277-026-07029-6 (PMC13128735; doi:10.1007/s00277-026-07029-6)
Supplement: Supplementary file 2 — Supplementary Material 2. [file 277_2026_7029_MOESM2_ESM.docx]

| Characteristics | WR-DLBCL  (n=33) | Extranodal OC-MR DLBCL  (n=43) | P value |
| --- | --- | --- | --- |
| R-CHOP | 29 | 32 | 0.244 |
| R-CHOP+X (IMiDs /BTKi) | 4 | 11 |  |
| auto-HSCT | 2 | 3 | 0.990 |
| non-transplant | 31 | 40 |  |
| Radiotherapy | 1 | 3 | 0.087 |
| non-Radiotherapy | 32 | 40 |  |

**Supplement Table 1. Comparison of treatment regimens between the two groups.**

Abbreviations: R-CHOP, rituximab, cyclophosphamide, doxorubicin, vincristine, and prednisone; IMiDs, immunomodulators; BTKi, Bruton's Tyrosine Kinase inhibitor; auto-HSCT, autologous stem cell transplantation.
