# Supplementary material for: A site-specific risk stratification model for extranodal diffuse large B-cell lymphoma in the oral cavity and maxillofacial region
Source: Ann Hematol. 2026 Apr 29;105(5):272. doi: 10.1007/s00277-026-07029-6 (PMC13128735; doi:10.1007/s00277-026-07029-6)
Supplement: Supplementary file 3 — Supplementary Material 3. [file 277_2026_7029_MOESM3_ESM.docx]

**Supplement Table 2. Univariate analysis of clinical and pathological prognostic factors in WR-DLBCL**

| **Variables** | **OS** | | | **PFS** | | |
| --- | --- | --- | --- | --- | --- | --- |
|  | **HR** | **95%CI** | **P** | **HR** | **95%CI** | **P** |
| **Ki67**  **>80% v ≤80%** | 1.723 | 0.287-10.329 | 0.552 | 2.311 | 0.270-19.803 | 0.445 |
| **CRP (mg/L)**  **>5.83 v ≤5.83** | 0.422 | 0.047-3.797 | 0.442 | 0.889 | 0.163-4.857 | 0.892 |
| **Alb (g/L)**  **<37 v ≥37** | 5.121 | 0.811-32.326 | 0.082 | 6.501 | 1.0-42.286 | 0.05 |
| **β2-MG (mg/L)**  **>2.18 v ≤2.18** | 1.114 | 0.725-1.713 | 0.621 | 0.949 | 0.579-1.556 | 0.836 |
